# Investigation of possible transmission of a susceptible microorganism through a contaminated duodenoscope; a case report

**DOI:** 10.1186/s13756-021-00996-7

**Published:** 2021-08-28

**Authors:** Judith A. Kwakman, Arjan W. Rauwers, Corné H. W. Klaassen, Marco J. Bruno, Margreet C. Vos

**Affiliations:** 1 Department of Gastroenterology and Hepatology, Erasmus MC University Medical Center, Dr. Molewaterplein 40, Mailbox 2040, Rotterdam, The Netherlands; 2 Department of Medical Microbiology and Infectious Diseases, Erasmus MC University Medical Center, Rotterdam, The Netherlands

**Keywords:** Duodenoscope, Duodenoscope-associated infection (DAI), Endoscopic retrograde cholangiopancreaticography (ERCP), Nosocomial infection, Case report

## Abstract

**Background:**

Despite compliance to extensive reprocessing protocols, duodenoscopes have been linked to outbreaks of susceptible and multi-drug resistant organisms (MDRO) due to persistent duodenoscope contamination. Duodenoscope-associated infections (DAIs) based on transmission of susceptible microorganisms are likely to be underreported due to detection bias.

**Case presentation:**

We describe the retrospective detection of a DAI case caused by a susceptible microorganism which at the time of clinical infection was not recognized as such. During 2017 and 2018, duodenoscopes were cultured on a daily basis due to research activities. While analyzing this data, it was found that a duodenoscope had been contaminated with *Enterobacter cloacae* complex over a period of 3 months. We checked whether patients treated with this duodenoscope had developed infections and found one patient with an *E. cloacae* cholangitis 3 months after the ERCP (Endoscopic retrograde cholangiopancreaticography) procedure. The isolates on the duodenoscope and in the patients’ blood culture were indistinguishable by amplified fragment length polymorphism (AFLP). By classical multi-locus sequence typing (MLST), both strains were of the same (but novel) sequence type. Application of whole genome MLST showed 93 (out of 3757) allelic differences.

**Conclusion:**

This case report describes a plausible link between a contaminated duodenoscope and a patient infection with *E. cloacae*. Transmission of susceptible *E. cloacae* was highly suspected from AFLP and MLST results; by WGS, 93 allelic differences were found which proves closely related strains. This report shows that DAIs by susceptible microorganisms can be easily missed and therefore its true prevalence remains underscored.

## Introduction

Duodenoscope-associated infections (DAI) are infections caused by microorganisms transmitted from contaminated duodenoscopes into patients undergoing an endoscopic retrograde cholangiopancreaticography (ERCP). Up until now, these kind of exogenous infections are known from outbreak reports, describing contaminated endoscopes serving as a source of transmission to one or more patients. At the end of the twentieth century, most reported outbreaks described the spread of susceptible gut microorganisms through improperly reprocessed duodenoscopes [1]. However, there has been a new surge of reported outbreaks in the past fifteen years. These are almost all based on multi-drug resistant organisms (MDRO) and have often occurred despite strict adherence to the manufacturer’s instructions for reprocessing [1]. The increase of DAI outbreaks by MDROs can partially be explained by an overall increase of MDRO prevalence [2].

It is assumed that DAIs, especially by susceptible microorganisms, are often mistaken for endogenous infections inherent to the ERCP procedure itself. Previous studies have shown post-ERCP bacteremia rates ranging between 0 and 27% [3]. However, no single study differentiated between endogenous and exogenous origins of bacteremia. Detecting and reporting bias hamper estimations of the true prevalence of DAIs, specifically of those caused by susceptible microorganisms. To date, no prospective studies investigated the prevalence of DAIs. Estimates are based mainly on outbreak reports and biased towards those caused by MDROs. Information on transmission of susceptible microorganisms is scarce, likely due to a low chance of alert and the complexity to detect and prove transmissions of susceptible microorganisms. MDRO are easier to recognize because they usually manifest as a cluster of infected cases unresponsive to standard treatment or detected through surveillance screening activities. This is contrary to cross infections caused by common susceptible microorganisms which in general respond quickly to standard antibiotic treatment and assumed to be caused by translocation of a patients’ own flora and thus categorized as endogenous post-ERCP infections [3, 4].

DAIs due to both MDRO and susceptible microorganisms are underreported because transmission goes undetected as endoscopes are not regularly cultured. In our center, we were able to use monthly surveillance cultures and an existing duodenoscope culture database to trace a DAI back to a specific duodenoscope. Between July 2017 and October 2018, all duodenoscopes and linear echoendoscopes (DLE) in the Erasmus MC (Rotterdam, the Netherlands) were cultured on a daily basis after high level disinfection (HLD) to evaluate whether adenosine triphosphate (ATP) monitoring after manual cleaning would result in lower contamination levels of patient-ready DLE. The personnel was blinded for the culture results during the study. After the study had ended, we noticed one duodenoscope had been repeatedly contaminated with *Enterobacter cloacae* complex over a period of 3 months. We decided to retrospectively investigate this period to assess whether this had led to transmission into patients, this resulted in the detection of one DAI case.

## Case presentation

**Fig. 1:**
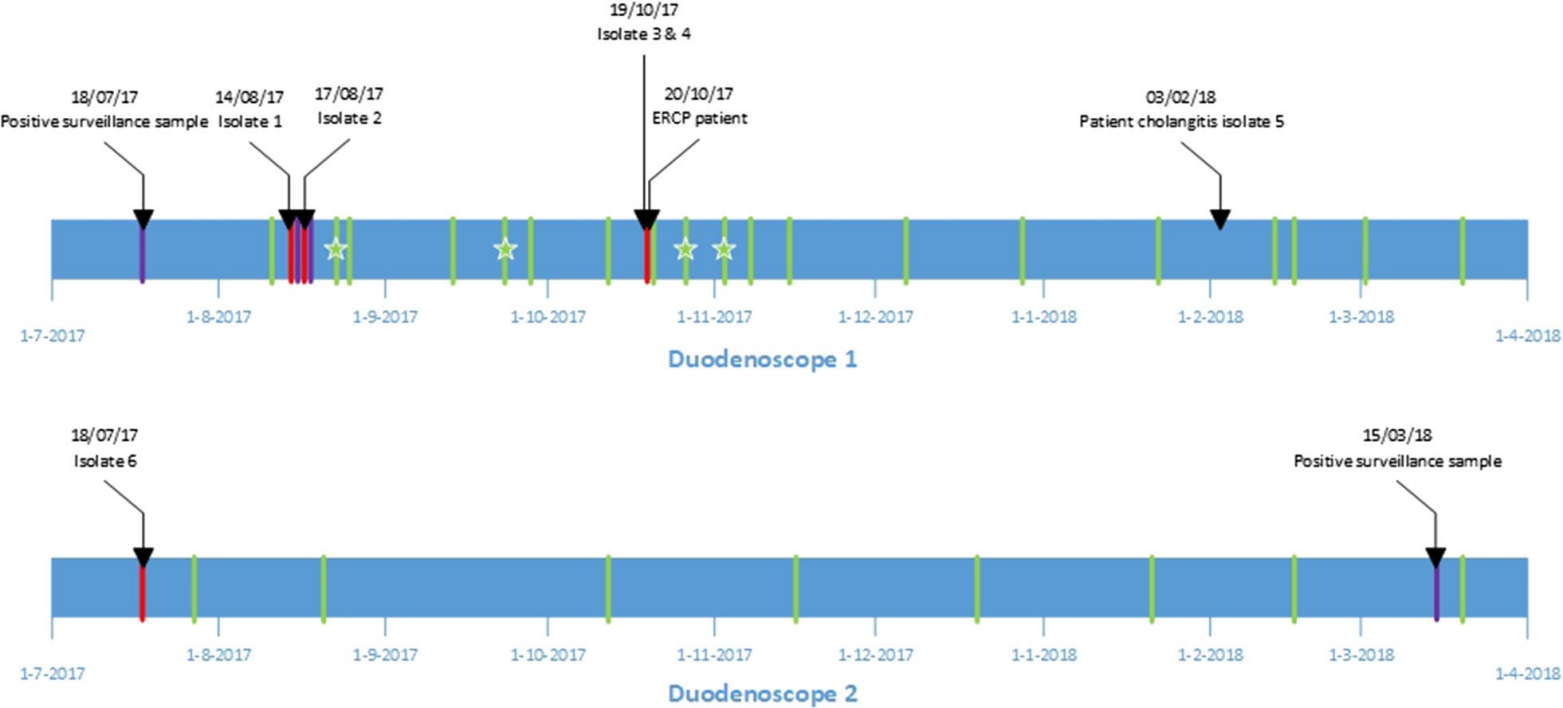
Timeline of contamination of duodenoscopes 1 and 2 and infection of patient A. Two different clusters of this microorganism are mentioned in this case study. Red stripes indicate duodenoscope samples positive for ECC cluster 1. Purple stripes indicate samples subjected to molecular typing but without a match with any other grown ECC. Green stripes indicate surveillance cultures negative for ECC, stars represent growth of *Klebsiella pneumoniae*. Only two of the ECC positive samples were acquired from the surveillance database, all other positive samples were derived from the study database

Duodenoscope 1 (ED34-i10T, Pentax, Dodewaard, The Netherlands) was found to be contaminated with *E. cloacae* complex on July 18th, August 14th, 15th, 17th and 18th and October 19th 2017. In our retrospective investigation of patients treated with duodenoscope 1 during the period of contamination, we came across a patient who developed an *E. cloacae* complex cholangitis three months after the ERCP procedure (Fig. 1). This patient underwent an ERCP with duodenoscope 1 at October 20th. At the time of the ERCP, the patient was treated with cetuximab for a rectal carcinoma with hepatic, pulmonary, and lymphogenic metastases. The patient was treated for this infection at an oncology ward for eleven days. Treatment included three times daily 1000 mg Meropenem antibiotics (Fresenius kabi Netherlands, Zeist, the Netherlands) for seven days and two ERCP procedures to replace a clogged stent. Furthermore, during the hospitalization, an X-thorax, abdominal echography and MRCP (magnetic resonance cholangio-pancreatography) were performed. The patient died six months after the infection due to progression of his malignant disease.

**Fig. 2:**
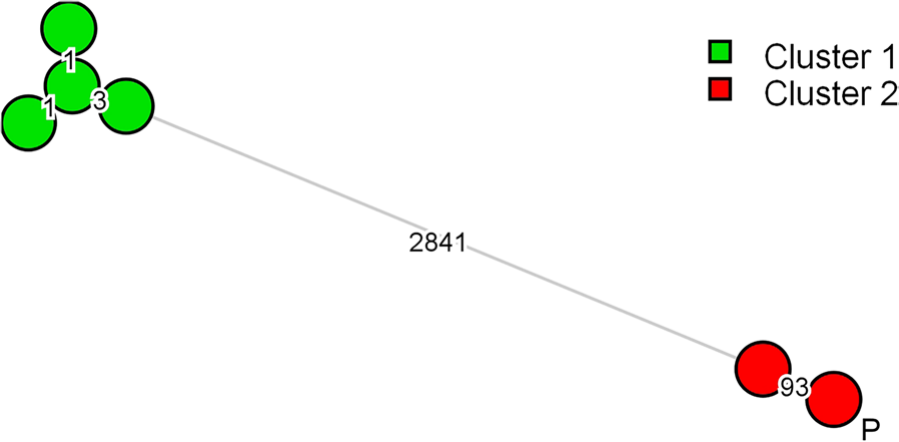
wgMLST typing of *E. cloacae* complex isolates. Minimum spanning tree based on a categorical analysis illustrating the relationship between the isolates from cluster 1 and cluster 2. Branch lengths indicate the number of allelic difference between isolates. The patient isolate is indicated with “P”

The stored *E. cloacae* complex isolates from the patients’ blood cultures and duodenoscope 1 were subjected to molecular typing to investigate relatedness. Initially, typing of these microorganisms was performed by amplified fragment length polymorphism (AFLP) using a combination of HpyCHIV4 + A and MseI + GT primers using established protocols [5]. Fingerprints were imported and analyzed using BioNumerics software (Applied-Maths, St-Martens-Latem, Belgium). Based on the obtained fingerprints, two clusters were identified, with the isolates from August 14th (isolate 1) and 17th (isolate 2) and October 19th (isolate 3) from duodenoscope 1 to belong to one cluster (ECC cluster 1). All isolates belonging to ECC cluster 1 were collected by brushing of the working channel of the duodenoscope (see Table 1 for specifics of all isolates). In the sample from the forceps elevator from October 19th, another *E. cloacae* complex isolate (isolate 4) was found as well that was different from the isolates in ECC cluster 1. This isolate and the isolate from the patients’ blood culture (isolate 5) were considered to be identical (ECC cluster 2) by AFLP (results not shown). Subsequently, both isolates were additionally subjected to whole genome sequencing (WGS) on an iSEQ platform (Illumina, San Diego, USA) using protocols recommended by the manufacturer. Paired-end reads (2x150 nt) were assembled using CLC Genomics Workbench (Qiagen, Hilden, Germany) and subsequently analyzed using the whole genome MLST (wgMLST) scheme available in BioNumerics (Applied-Maths, St-Latem-Martens, Belgium) providing both wgMLST based typing results as well as the conventional MLST sequence type. Isolates from ECC cluster 1 were of the same but novel sequence type (allelic profile 51-4-14-4-39-4-25, a dual locus variant (DLV) of ST134. Isolates from ECC cluster 2 were also of the same but another novel sequence type (allelic profile 59-9-6-485-62-83-6, a DLV of ST1283). Application of the wgMLST scheme showed up to three allelic differences between the isolates from ECC cluster 1, but 93 allelic differences, out of 3757, between the two isolates from ECC cluster 2 (Fig. 2). As *Enterobacter* species are still relatively unexplored using wgMLST, no cut off values have been proposed to define relatedness between members of *E. cloacae* complex. If the 93 allelic differences were located in a relatively small genomic region, these differences could have resulted from a single genetic event, increasing the likelihood that these strains are closely related. And although some of the divergent loci were indeed located in the same genomic contig, multiple contigs were involved indicating multiple genetic events (results not shown).

**Table 1:** Details of the six *E. cloacae* complex isolates found in the two duodenoscopes and blood cultures of the patient

	Isolate 1	Isolate 2	Isolate 3	Isolate 4	Isolate 5	Isolate 6
Date	14/08/17	17/08/17	19/10/17	19/10/17	03/02/18	18/07/17
Source	Duodenoscope 2	Duodenoscope 2	Duodenoscope 2	Duodenoscope 2	Patient	Duodenoscope 1
Sample site	Channel (brush)	Channel (brush)	Channel (brush)	Forceps elevator	Blood culture	Channel (brush)
Cluster	ECC cluster 1	ECC cluster 1	ECC cluster 1	ECC cluster 2	ECC cluster 2	ECC cluster 1
Bacterial quantity	> 100 CFU	10 CFU	8 CFU	90 CFU	2 positive blood culture bottles	4 CFU
WGS species	*E. hormaechei* subsp. *hoffmannii*	*E. hormaechei* subsp. *hoffmannii*	*E. hormaechei* subsp. *hoffmannii*	*E. hormaechei* subsp. *Steigerwaltii*	*E. hormaechei* subsp. *Steigerwaltii*	*E. hormaechei* subsp. *hoffmannii*
*Antibiogram*						
MIC meropenem	≤ 0.25	≤ 0.25	≤ 0.25	≤ 0.25	≤ 0.25	≤ 0.25
MIC imipenem	≤ 0.25	≤ 0.25	≤ 0.25	≤ 0.25	≤ 0.25	≤ 0.25
MIC gentamicin	≤ 1	≤ 1	≤ 1	≤ 1	≤ 1	≤ 1
MIC tobramycin	≤ 1	≤ 1	≤ 1	≤ 1	≤ 1	≤ 1
MIC cotrimoxazol	≤ 1	≤ 1	≤ 1	≤ 1	≤ 1	≤ 1
MIC ciprofloxacin	≤ 0.25	≤ 0.25	≤ 0.25	≤ 0.25	≤ 0.5	≤ 0.25
MIC colistin	≤ 0.5	≤ 0.5	≤ 0.5	≤ 0.5	≤ 0.5	≤ 0.5
MIC fosfomycin	≤ 16	≤ 16	≤ 16	≤ 64	≤ 64	≤ 16

*CFU* colony forming units

While investigating this case, we also noticed the working channel of another duodenoscope (TJF-160VR, Olympus, Zoeterwoude, The Netherlands) (duodenoscope 2) to have been contaminated with *E. cloacae* complex. This duodenoscope was found to be contaminated with *E. cloacae* complex only on July 18th 2017. This isolate (isolate 6) was found through AFLP and wgMLST to belong to ECC cluster 1 as well. No other duodenoscopes were contaminated with *E. cloacae* complex.

Eight months after the first positive duodenoscope culture, duodenoscope 2 was again tested positive through the surveillance system for *E. cloacae* complex but this strain was different from all previously found *E. cloacae* complex isolates. All other surveillance cultures of duodenoscope 2 between July 2017 and March 2018 were negative for growth of gut microorganisms. Duodenoscope 1 never tested positive again for *E. cloacae* complex after October 19th 2017, and was replaced by a new model at the end of March 2018. Due to four positive surveillance cultures for growth of *Klebsiella pneumoniae* in the period August-November 2017, duodenoscope 1 was more often subjected to surveillance sampling than duodenoscope 2.

Investigation of this case was approved by the local ethical committee (METC number MEC-2019-0807).

## Discussion

This study describes the retrospective detection of a DAI caused by a normal susceptible microorganism and two duodenoscopes contaminated with the same microorganism. Due to the retrospective nature of this study, the exact route of transmission between the two duodenoscopes could not be clarified. This ERCP center uses a more extensive surveillance system than the twice a year surveillance prescribed by national guidelines to detect contaminated duodenoscopes [6]. Duodenoscope surveillance cultures were collected monthly and in the case of duodenoscope 1, even three times a month during the contamination period. However, even despite this more intense surveillance, contamination of the described duodenoscopes was missed. The study cultures revealed at least five moments duodenoscope 1 was contaminated with *E. cloacae* complex that were missed by surveillance. This DAI would only have been avoided if this duodenoscope was cultured on a daily basis while being quarantined awaiting culture results.

In recent years, a higher level of awareness regarding contaminated duodenoscopes and subsequent serious infections was noticed as demonstrated by literature reports of multiple incidents [1]. However, DAIs are underreported as this needs elaborate and specific microbiological culture and surveillance actions. For improvement three prerequisites are needed. First, cultured microorganisms of endoscopes and patients need to be stored. Second, linking patient cultures to contaminated duodenoscopes should be easier, for instance with alerts in the electronic patient files in case a used duodenoscope is found to be contaminated. Third, if a match is suspected, molecular typing should be performed to prove indistinguishability of the microorganisms found in the patient and duodenoscope cultures. In this case, we started with molecular typing using AFLP, which is a relatively quick and affordable typing method. Only the isolates that were indistinguishable through AFLP were subjected to WGS. WGS is a more laborious and expensive method, but provides a more in-depth analysis of the genetics of the different microorganisms [7]. In most DAI outbreak investigations, molecular typing is performed using PCR techniques [8–10]. Only in a few outbreaks, WGS was used to distinguish the microorganisms found on the duodenoscopes and in the affected patients [11–13]. WGS is superior to other types of molecular typing such as AFLP or pulsed-field gel electrophoresis (PFGE) in strain typing [7, 14].

In this report, AFLP and classical MLST results showed the microorganism in the patients’ blood culture to be indistinguishable from isolate 4 grown from duodenoscope 1. They were found to be of a new MLST type, making transmission of the same type very plausible. However, WGS of the two microorganisms revealed 93 allelic differences in the 3757 identified alleles. There is no specific cut-off level to determine if both microorganisms could still be related despite these allelic differences [15]. Thresholds of maximum differences in WGS typing are species specific and are not clearly defined. In clinical practice, cut-off levels are usually set at ~  < 1% of the analyzed genes. However, predetermined cut-off values need to be used cautiously as they do not take into account situation specific characteristics. This includes the long interval between the acquisition of the samples from the duodenoscope and the patient, the method of storage of the distinct isolates and the disinfection of the duodenoscope prior to sampling. These characteristics might have influenced the WGS results of the specific microorganism. Also, although 93 different alleles sound like a large difference, it is not much compared to truly unrelated isolates. Upon analysis of other *E. cloacae* complex isolates from various origin, isolates that were considered related or unrelated showed either < 5 or > 2000 allelic differences, respectively (results not shown). The 93 allelic differences, together with the observation that these isolates represented yet unknown MLST types indicate a certain degree of relatedness and that these isolates might link to a common source. Therefore, in our case, WGS did not provide conclusive proof of a transmission, but all results taken together with the epidemiological information makes it plausible that there is a link between the contaminated duodenoscope and the patient infection.

Several factors explain why this DAI was not considered and recognized as such at the time of infection. First, the long time period of 106 days between the ERCP procedure and infection likely contributed to the fact that this infection was not recognized as a DAI in clinical practice. Second, as this infection was caused by a susceptible common gut microorganism, which is not a rare cause of cholangitis [16], it is understandable that the treating physicians did not consider the duodenoscope to be the source. Third, due to the patient’s underlying malignancy, he was treated on an oncology instead of a gastroenterology ward. Awareness regarding the possible occurrence of DAIs is likely to be lower or even absent among non-gastroenterological personnel compared to health care workers involved in ERCPs on a daily basis. Fourth, the microorganisms of interest were found in study cultures for which clinicians and infection prevention practitioners were blinded per study protocol. Concurrent surveillance cultures did not detect *E. cloacae* complex or other gut flora. This shows that monthly surveillance cultures are inadequate to detect DAIs.

No patients were found to be treated with both duodenoscopes in the period between the first confirmed contamination of duodenoscope 1 and 2, making it unlikely that one patient was responsible for the contamination of both duodenoscopes. Therefore, a contamination source in the reprocessing surroundings could explain how these two separate duodenoscopes became contaminated with the same microorganism. However, since our investigation took place long after the transmissions had occurred, no environmental samples were acquired to test this hypothesis. Cultures of the final rinse water of the automated endoscope reprocessors (AERs) collected in July and October were negative for growth of any microorganisms. No other duodenoscopes were contaminated with this specific strain. Both duodenoscopes had recently been investigated by the manufacturer. Duodenoscope 1 was sent to the manufacturer on August 9th to check an issue with the image quality, duodenoscope 2 was sent to the manufacturer on July 14th for periodic maintenance. Hypothetical explanations for a transmission between the two duodenoscopes could be that any of the cleaning materials (i.e. brushes or syringes) were used on both duodenoscopes instead of being disposed after every use or that the reprocessing staff served as a transmission vector due to incorrect use of protective equipment or incidental breaches in the reprocessing protocol. However, since the transmission took place during a study which was focused on reprocessing efficacy, it seems more likely that the reprocessing staff adhered very strictly to the cleaning protocols in this period.

Duodenoscope 1 contained two different strains of *E. cloacae* complex prior to the ERCP of the patient. The strain belonging to ECC cluster 1 was found in three samples of the working channel collected over a period of 2 months. The strain belonging to ECC cluster 2 was identified once and only found in a sample from the forceps elevator. Our hypothesis is that the strain from ECC cluster 1 was able to develop a biofilm in the channel, explaining its persistence over 2 months, whereas the presence of the strain from ECC cluster 2 was more likely to be an incidental event.

In 2012, our hospital was affected by a duodenoscope-associated outbreak which caused infections with a VIM-2 producing *Pseudomonas aeruginosa* in eight patients [17]. Ever since, we have been actively involved in research regarding duodenoscope contamination and DAIs. The current DAI experience has led to a few local changes in our hospital, such as the use of different brushes, borescope inspections and an intensified surveillance protocol. In other outbreak reports, the outbreaks were ended by more strict adherence to reprocessing and drying protocols [10], by removal of the duodenoscopes [18, 19] or by introducing ethylene oxide sterilization [19–21]. In our case, the contamination of duodenoscope 1 disappeared spontaneously after 3 months and the duodenoscope was already removed from service at the time of this retrospective investigation. However, since duodenoscope contamination remains a worldwide problem with approximately 15% of all patient-ready duodenoscopes being contaminated [22], structural solutions are needed to ultimately banish this problem. Ever since the first outbreak reports, a lot has changed in the reprocessing methods, with the introduction of AERs and drying cabinets. Also, several national and international guidelines have been developed over the years and duodenoscope manufacturers have altered the duodenoscope designs to improve cleaning accessibility [23]. The FDA has been actively involved in the topic since 2013 and has issued numerous recommendations and warnings on the use and reprocessing methods of duodenoscopes and has ordered the three main duodenoscope manufacturers to conduct postmarket surveillance studies [24]. Despite all this effort, a zero contamination rate has not yet been reached for reusable duodenoscopes. The only realistic option to completely prevent DAIs is switching to the recently introduced disposable duodenoscopes for single use. However, the substantial costs associated with that switch make it unlikely that reusable duodenoscopes will soon be replaced by disposable ones [25].

Although treatment of endogenous and exogenous post-ERCP infections are not necessarily different from one another, it is important to make this distinction. DAIs are preventable, but rare iatrogenic complications, whereas endogenous infections are in general inherent to the ERCP procedure. Since patients undergoing ERCP procedures are often vulnerable and immunocompromised, such as our patient described here, post-ERCP infection is a serious and potentially fatal complication [19]. Furthermore, one contaminated duodenoscope can remain contaminated over a long period of time and infect multiple patients [9, 10, 20, 26], as is demonstrated by outbreak reports in which 12–41% of the exposed patients became colonized [27, 28]. The sooner the duodenoscope is identified as the source of transmission, the more patients can be excluded from undergoing treatment with this contaminated duodenoscope and hence the risk of becoming infected.

Currently, some guidelines advise to perform microbiological surveillance periodically, but there is no consensus on the intervals. European guidelines [29] recommend surveillance at least every 3 months, Australian guidelines [30] recommend monthly surveillance, the American Society for Gastrointestinal Endoscopy does not urge periodic surveillance [31]. This case demonstrates that even more than once a month sampling can miss intermittent contamination and might create a false impression of safety. Only a culture-and-hold-method can fully prevent contaminated duodenoscopes from being used in ERCPs [32]. However, this requires an investment in extra duodenoscopes and in daily microbiological testing, which is not feasible in most endoscopy centers.

It is important to share information on DAI incidence and local reprocessing issues globally to stimulate awareness and the development of solutions. In a 2016 US senate report, it was mentioned that the FDA and CDC are only rarely actively notified by hospitals and endoscope manufacturers on these issues [33]. This has led to an official warning towards the three main duodenoscope manufacturers [34]. Timely reporting of incidents to the authorities, will sooner lead to actions protecting patients all over the world.

## Conclusion

In conclusion, this report shows that DAIs by susceptible microorganisms can occur unnoticed and more research is required to estimate its prevalence. Epidemiologic information, bacterial identification, susceptibility testing and AFLP point to a link between the contaminated duodenoscope and the infection in the patient. Unfortunately, the WGS results cast doubt on this conclusion. Notwithstanding the WGS results, we feel that this case points to a possible role of duodenoscopes as routes of transmission.

## Data Availability

All data generated or analyzed during this study are included in this published article.
